# Veno-venous ECMO indications: more than respiratory support

**DOI:** 10.1186/s13054-019-2555-x

**Published:** 2019-08-07

**Authors:** Aaron Blandino Ortiz, David Cabestrero Alonso, Raúl De Pablo Sánchez

**Affiliations:** Intensive Care Department, Hospital Universitario Ramón y Cajal, Ctra. Colmenar Viejo, KM 9100, 28034 Madrid, Spain

Dear Editor.

Recently, we have read with tremendous interest the article published by Prof. Gattinoni et al. [1], which is focused on certain aspects that are under-evaluated in extracorporeal gas exchange therapy.

Obviously, veno-venous extracorporeal membrane oxygenation (VV ECMO) indications are related to respiratory syndromes, and traditionally, its indication criteria were limited to gas exchange and respiratory mechanics. However, we consider that VV ECMO indications should not be limited to these. Cardiopulmonary interactions during severe acute respiratory distress syndrome (ARDS), perhaps, plays a paramount role in the pathophysiologic derangements which leads to organic failure with its obvious consequences, from the increase in right ventricle (RV) afterload secondary to hypoxic pulmonary vasoconstriction [1] to the increase in intrathoracic pressure (ITP) (related to the syndrome and induced by mechanical ventilation) which affects pressure gradients for both, systemic venous return to the RV and systemic outflow from the left ventricle (LV) [2]. It has been found that cardiovascular abnormalities are more implied in the presumed cause of death in the PROSEVA trial, where only 15% of the patients had hypoxemia as a presumed cause of death, in counterpart to 21% and 49% related to refractory shock and multiple organ dysfunction [3]. As in the post hoc analysis of mortality at day 28, the supine group in the quartile with PaO_2_/FiO_2_ between 124 and 150 had a mortality rate of 35%. Moreover, this favorable cardiovascular effect could explain the clinical benefits of prone positioning in terms of mortality reduction, thanks to the decrease in afterload and unloading of the RV [4, 5].

We do not deny the clinical importance of hypoxemia in the pathophysiological context of ARDS as a VV ECMO indication, but certainly a more realistic approach should look beyond the classical criteria to start ECMO therapy and take into account the heart-lung interactions its effects and implications in the prognosis of patients with acute respiratory failure. Also, we understand that more data and scientific evidence is needed to support such clinical approach, but perhaps it is time to start raising it and consider it as a potential indication in future clinical studies.

## Data Availability

Not applicable.

## References

[1] Gattinoni L, Vassalli F, Romitti F, et al. Extracorporeal gas exchange: when to start and how to end? Crit Care. 2019;23(S1). 10.1186/s13054-019-2437-2. 10.1186/s13054-019-2437-2 PMC6570632 31200746

[2] Pinsky M (2018). Cardiopulmonary interactions: physiologic basis and clinical applications. Ann Am Thorac Soc.

[3] Guérin C, Reignier J, Richard J (2013). Prone positioning in severe acute respiratory distress syndrome. N Engl J Med.

[4] Vieillard-Baron A, Charron C, Caille V (2007). Prone positioning unloads the right ventricle in severe ARDS. Chest.

[5] Jozwiak M, Teboul JL, Anguel N (2013). Benefcial hemodynamic effects of prone positioning in patients with acute respiratory distress syndrome. Am J Respir Crit Care Med.

